# Effects of experimental design on calibration curve precision in routine analysis

**DOI:** 10.1155/S1463924698000029

**Published:** 1998

**Authors:** Maria Fernanda Pimentel, Benício de Barros Neto, Teresa Cristina B. Saldanha, Mário César Ugulino Araújo

**Affiliations:** 1 Instituto Tecnológico de Pernambuco-ITEP PE Brazil; 2 Departamento de Química Fundamental-CCEN Universidade Federal de Pernambuco Brazil; 3 Departamento de Química-CCEN Universidade Federal da Paraíba João Pessoa PB 58.051-970 Brazil

## Abstract

A computational program which compares the effciencies of different experimental designs with those of maximum precision (D-optimized designs) is described. The program produces confidence interval plots for a calibration curve and provides information about the number of standard solutions, concentration levels and suitable concentration ranges to achieve an optimum calibration. Some examples of the application of this novel computational program are given, using both simulated and real data.


Journal of Automatic Chemistry, Vol. 20, No. 1 (January–February 1998) pp. 9–15

### Effects of experimental design on calibration curve precision in routine analysis

**Maria Fernanda Pimentel,**
*Instituto Tecnológico de Pernambuco-ITEP-PE-Brazil*

**Benício de Barros Neto,**
*Departamento de Química Fundamental-CCEN-Universidade Federal de Pernambuco*

**Teresa Cristina B. Saldanha and Mário César Ugulino Araújo***

*Departamento de Química-CCEN-Universidade Federal da Paraíba 58.051-970-João Pessoa-PB-Brazil*

*A computational program which compares the efficiencies of different experimental designs with those of maximum precision (D-optimized designs) is described. The program produces confidence interval plots for a calibration curve and provides information about the number of standard solutions, concentration levels and suitable concentration ranges to achieve an optimum calibration. Some examples of the application of this novel computational program are given, using both simulated and real data.*

### Introduction

Calibration is a very important step in any analytical procedure. The choice and arrangement of standard solutions, i.e. experimental design, may affect the precision with which a calibration curve can be estimated. Since a calibration curve is the basis for predicting concentrations of unknown samples, the purpose of good experimental design is to obtain the best possible predictive power [1].

Although the theoretical importance of experimental design is widely recognized [2], the relevance of its applicability to analytical laboratory procedures has only attracted limited attention [3, 4]. For the construction of a calibration curve, the usual practice is still to divide the experimental region uniformly. This procedure, however, is only indicated when there is a need to establish the linear concentration range. For routine analyses, where this range is known in advance, this procedure is not recommended.

This paper describes a computational program which allows selection of the best experimental design for a given situation, based on efficiency values and plots of confidence intervals. Some examples of its application in typical routine analysis are also given, using simulated and real data.

* Author to whom correspondence should be addressed.

### Theory

The underlying theoretical principles used in this paper follow; a more detailed discussion can be found elsewhere [5–7].

Assuming the linear model, $y = b_0 + b_1 x$ (where $y$ is the dependent variable, $x$ the independent variable and $b_0$ and $b_1$ the estimates of the model parameters), the precision of the calibration curve can be evaluated from the width of the confidence interval for the analytical response $y_0$ corresponding to a certain concentration value, $x_0$, which is given by:

$$(y_0)_e = y_0 \pm ts\left[1 + \frac{1}{n} + \frac{(x_0 - x_m)^2}{\Sigma(x_i - x_m)^2}\right]^{1/2} \qquad (1)$$

where $(y_0)_e$ is the true value of the analytical response at concentration $x_0$; $y_0$ is the predicted response by the model for the concentration $x_0$; $t$ is a point in Student's distribution; $s$ is the estimate of the standard deviation of $y$; $n$ is the number of measured responses; and $x_m$ is the average of $x$ values.

In matrix notation, this equation can be written as:

$$(y_0)_e = y_0 \pm ts[1 + \mathbf{x}_0'(\mathbf{X}'\mathbf{X})^{-1}\mathbf{x}_0]^{1/2} \qquad (2)$$

where:

$$\mathbf{X} = \begin{bmatrix} 1 & x_{11} & \dots & x_{1k} \\ 1 & & & \\ \vdots & \vdots & & \vdots \\ 1 & x_{n1} & \dots & x_{nk} \end{bmatrix}$$

$$\mathbf{x}_0 = \begin{bmatrix} 1 \\ x_1 \\ \vdots \\ x_k \end{bmatrix}$$

$n$ is the number of measured responses and $k$ is the number of independent variables (in the linear model $k = 1$).

Analytical chemists will primarily use the calibration curve to predict the value of the independent variable (concentration), $x_0$, corresponding to a specific value of the analytical response, $y_0$. This procedure is called 'inverse regression'. The confidence intervals around the estimated concentration, $(x_0)_e$, are given by equation (3), which is similar to equation (2). The only difference between the two equations is the appearance of the term

0142-0453/98 $12·00 © 1998 Taylor & Francis Ltd

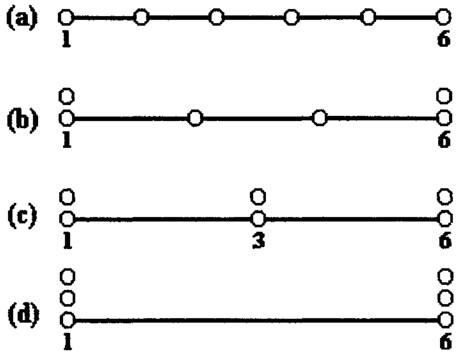

*Figure 1. Four designs for the same range, with the same number of measurements.*

$b_1$ in equation (3), which represents the straight line angular coefficient:

$$(x_0)_e = x_0 \pm \frac{ts}{b_1}[1 + \mathbf{x}_0'(\mathbf{X}'\mathbf{X})^{-1}\mathbf{x}_0]^{1/2} \qquad (3)$$

Analysis of equations (2) and (3) reveals that the confidence interval depends on the experimental design defined by the matrix $(\mathbf{X}'\mathbf{X})^{-1}$, which is square with dimension $p$ ($p$ is the number of parameters in the calibration model). The best design, as far as precision is concerned, is the one that somehow minimizes this matrix. Among the several minimization criteria already proposed, the most popular consists in choosing the design that minimizes the determinant of $(\mathbf{X}'\mathbf{X})^{-1}$. Such a design is said to be D-optimized [3, 8].

For D-optimized designs, the number of concentration levels must always be equal to the number of parameters in the chosen model. For a linear model, for example, which is defined by two parameters, the minimum value of $\det(\mathbf{X}'\mathbf{X})^{-1}$ is attained when the concentration levels coincide with the two extreme points of the calibration range. In practice, D-optimized designs are not recommended for all situations, as sometimes it is not previously known whether the functional relationship between $x$ and $y$ is really linear within the range of interest. In such cases, the experimental design must include at least three concentration levels, to allow for a lack-of-fit test of the proposed model [6].

For a given calibration range and a number of measurements, several different designs are possible. Figure 1 shows four designs, all with the same number of measurements and the same working range (1–6 arbitrary concentration units). Design a is often employed in routine laboratories. Design d is the D-optimized design for these conditions, and results in the most precise calibration curve. A way of comparing a given design with the corresponding D-optimized design is to calculate its efficiency, which is defined by equation (4):

$$E = \frac{\det(\mathbf{X}'\mathbf{X})}{\det(\mathbf{X}'\mathbf{X})_{\text{D-otim}}} \cdot 100 \qquad (4)$$

where $\det(\mathbf{X}'\mathbf{X})$ is the determinant of $(\mathbf{X}'\mathbf{X})$ for the chosen design and $\det(\mathbf{X}'\mathbf{X})_{\text{D-otim}}$ is the determinant of $(\mathbf{X}'\mathbf{X})$ for the D-optimized design with the same number of measurements.

The computational program 'DESIGN', presented in this paper, provides values for efficiencies and plots of confidence intervals, with a view to helping analytical chemists to decide which experimental design is most suited to a given situation.

### *Description of the DESIGN computational program*

DESIGN was written in the MATLAB high-level language and was designed to be user-friendly; a flow chart of the program is shown in figure 2. Three options allow evaluation of different experimental designs in fitting linear models:

(1) Selection of the number of standard solutions, taking into account the width of the confidence interval for the D-optimized design.
(2) Selection of the concentration levels (number and location).
(3) Comparison of several designs.

In option 1, the user evaluates the effect of the number of standard solutions used for calibration, from plots of confidence intervals for D-optimized designs. The program asks for the lower and higher concentrations of the working range, and the number of standard solutions of the design.

Once the number of standard solutions is chosen, option 2 permits the effect of the number and distribution of concentration levels on the calibration curve to be estimated. Efficiencies and plots of confidence intervals for selected designs are given. Since this option is intended to access only the effect of how many and which levels should be selected, all designs must have the same lower and higher concentrations, in addition to the same number of standard solutions.

In option 3, there is no constraint on the designs under comparison. Designs with different numbers of standard solutions, different numbers of concentration levels or different working ranges are evaluated through confidence interval plots. The concentration range shown on the plot is chosen by the user.

The subprogram used to calculate confidence intervals and efficiencies is described in listing 1 in the Appendix to this paper.

### Simulations

Two practical examples were used to demonstrate the use of the options in the DESIGN program: the determination of potassium and iron in drinking water by flame emission spectroscopy (FES) and inductively-coupled plasma atomic emission spectrometry (ICP-AES), respectively. For both cases a calibration curve needs to be constructed relating the intensity of the measured signal (dependent variable $y$) to the concentration of the analyte in the sample (independent variable $x$).

It is important to bear in mind that replicate levels require full authentic replicate determinations, and not just replicated measurements of the same solution.

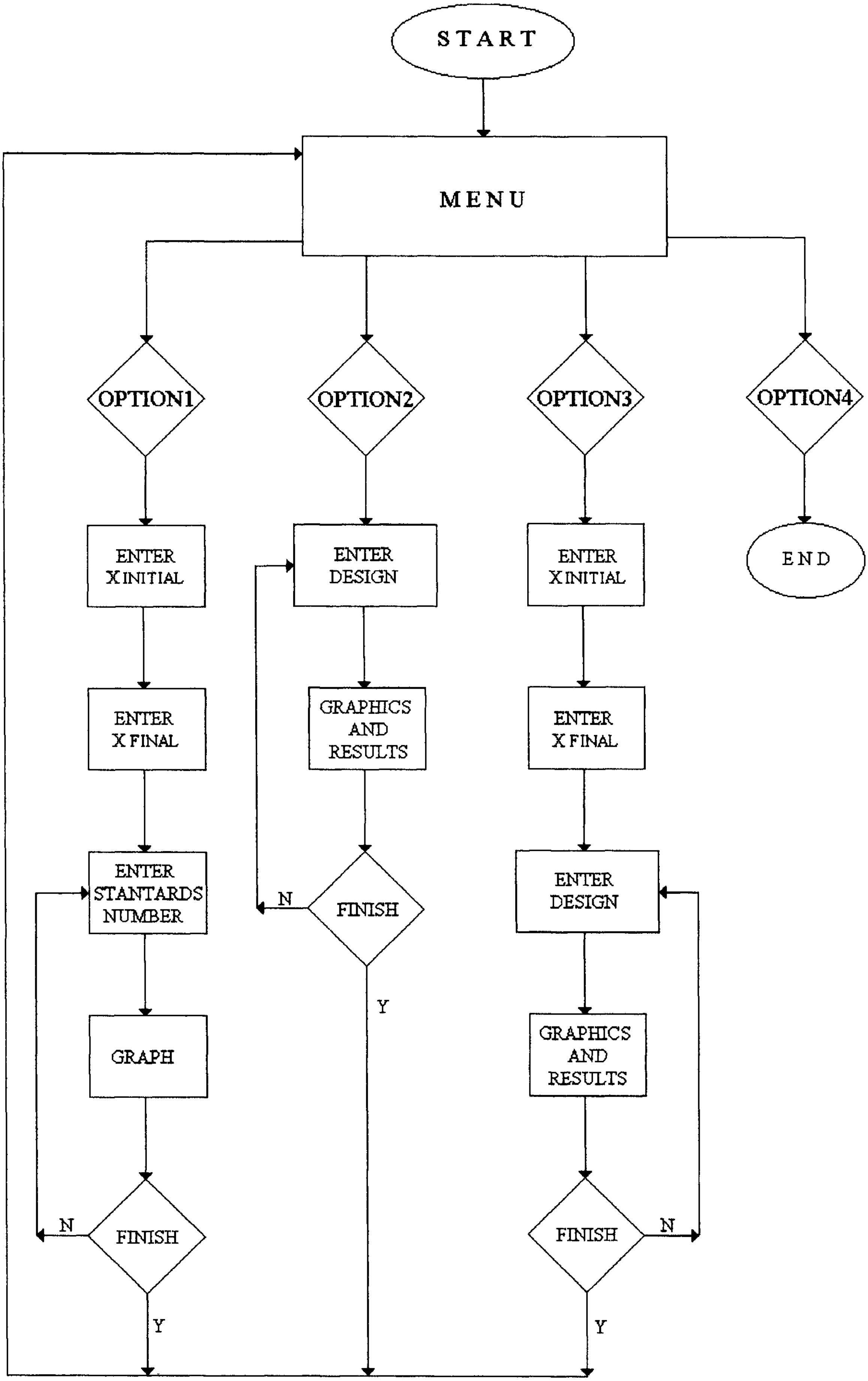

*Figure 2. Flow chart of the 'DESIGN' program.*

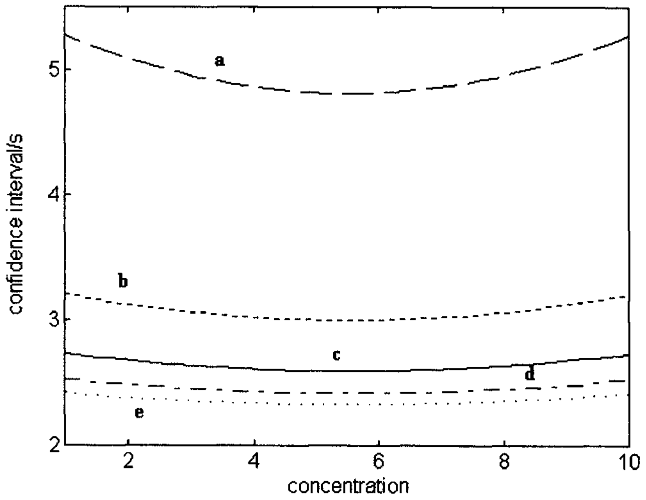

*Figure 3. Comparison of designs with different number of standard solutions* ($N$); (a) $N = 4$, (b) $N = 6$, (c) $N = 8$, (d) $N = 10$, (e) $N = 12$.

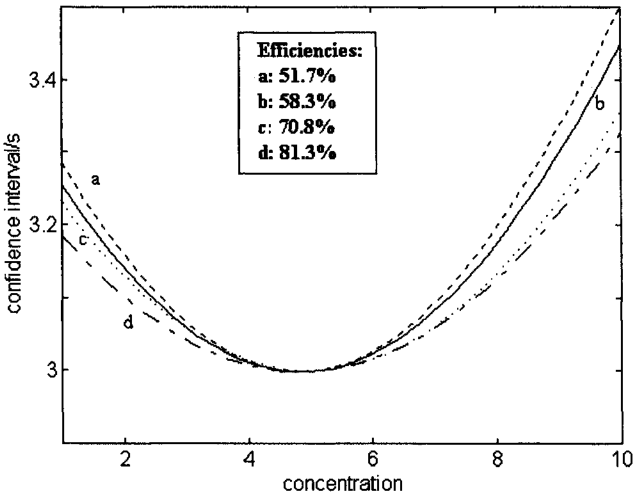

*Figure 4. Comparison of designs with different number of concentration levels; (a) design* [1 2 3 5 8 10], *six levels; (b) design* [1 1 3 6 8 10], *five levels; (c) design* [1 1 3 5 10 10], *four levels; (d) design* [1 1 1 6 10 10], *three levels.*

*Selection of the number of standard solutions (option* 1)

Assuming a linear relationship within the working range, the number of standard solutions to be used in a calibration curve can be chosen from observation of the plots generated by the program in option 1.

In the determination of potassium by FES, the known linear range is between 1 and 10 mg/l. In order to demonstrate the influence of the number of standard solutions on the precision of the fitted calibration curve, the following designs were chosen:

a: [1 1 10 10], $N = 4$

b: [1 1 1 10 10 10], $N = 6$

c: [1 1 1 1 10 10 10 10], $N = 8$

d: [1 1 1 1 1 10 10 10 10 10], $N = 10$

e: [1 1 1 1 1 1 10 10 10 10 10 10], $N = 12$.

Figure 3 shows that the larger the number of standard solutions, the smaller the confidence interval and, therefore, the more precise the calibration curve. This is explained by the decrease in $t$ and $1/n$ in equation (1). However, it is also evident that this trend is progressively damped, so that beyond a certain point (e.g. $N > 6$) the improvement in precision does not seem to be enough to compensate for the additional effort of preparing more standard solutions. It is then suggested that designs with six different standard solutions are adequate for the construction of calibration curves for routine analysis.

*Selection of the number of concentration levels (option* 2)

For a linear working range, the ideal experimental design is the D-optimized one [3, 8]. In practice, however, range linearity cannot always be taken for granted and it is advisable to employ at least three concentration levels to allow for a lack-of-fit test of the assumed linear model [5–7]. The selection of how many and which concentration levels are to be used to construct the calibration curve can be carried out through comparison of the efficiencies of the chosen designs with that of the D-optimized design. In potassium determination by FES, for example, problems in nebulization may change the linear range and therefore a periodic linearity test is recommended.

In order to select the number of concentration levels the following designs may be considered:

a: [1 2 3 5 8 10], six levels b: [1 1 3 6 8 10], five levels c: [1 1 3 5 10 10], four levels d: [1 1 1 6 10 10], three levels.

Decreasing the number of levels increases the design efficiency and consequently improves the precision of the calibration curve too (see figure 4). Nevertheless, this tendency cannot be generalized, because design efficiency depends not only on the number of levels but also on which points in the working range they are located at. To illustrate this, let us consider the following designs, whose efficiencies are shown in figure 5:

a: [1 1 5 5 10 10], three levels b: [1 1 3 7 10 10], four levels c: [1 1 2 9 10 10], four levels.

As shown in figure 5, although design a has only three levels, it results in lower efficiency relative to the other four-level designs. Thus, the selection of concentration levels can be very specific and highly dependent on the kind of analysis being performed. In general, it is recommended that:

(1) Four concentration levels be used, to have not only high efficiency but also two degrees of freedom to test for lack of fit of the linear model [4, 5].
(2) Measurements be taken as close as possible to the calibration range limits, which is the choice associated with D-optimized designs.

From these considerations, an experimental design like c in figure 5 should be employed in the example of the FES potassium assay in water. This design involves six

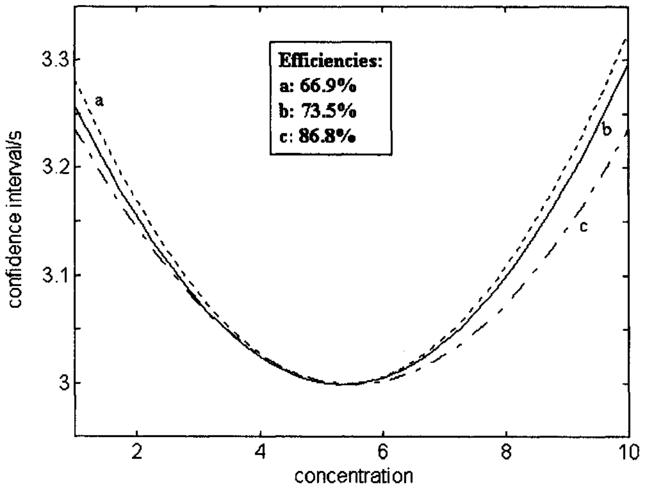

*Figure 5. Comparison of designs with different number of concentration levels; (a) design* [1 1 5 5 10 10], *three levels; (b) design* [1 1 3 7 10 10], *four levels; (c) design* [1 1 2 9 10 10], *four levels.*

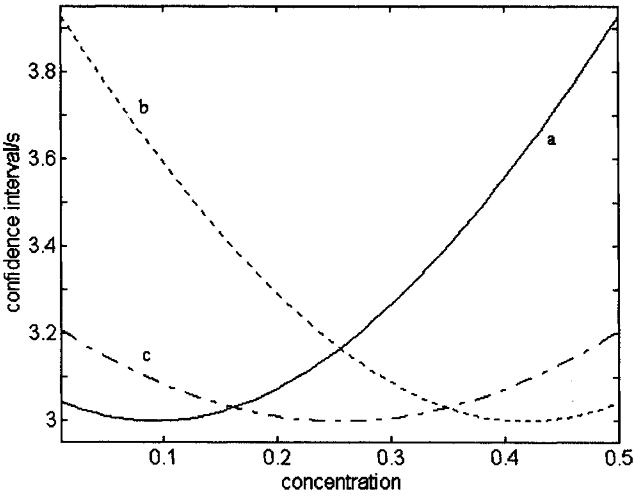

*Figure 6. Comparison of designs with different concentration means;* (a) *design* [0.01 0.01 0.01 0.01 0.01 0.5], mean = 0.09 mg/l; (b) *design* [0.01 0.5 0.5 0.5 0.5 0.5], mean = 0.42 mg/l; (c) *design* [0.01 0.01 0.01 0.5 0.5 0.5], mean = 0.25 mg/l.

standard solutions distributed in four concentration levels and allows for lack-of-fit testing with two degrees of freedom, yet still has a high efficiency.

*Comparison between designs (option* 3)

Consider, for example, the determination of iron in drinking water by ICP-AES. In most of the samples, iron concentration is between 0.01 and 0.5 mg/l and this working range is normally used, although the linear range is much larger. As a consequence, it is not necessary to test for linearity. Besides, it is known from experience that iron concentration in uncontaminated drinking waters is close to 0.01 mg/l, rising to 0.5 mg/l in iron-contaminated samples. Which design should be adopted for analysing these samples? Recalling that the precision of a calibration curve increases towards the mean [term $(x_0 - x_m)$ in equation (1)], designs like the following can be suggested:

a: [0.01 0.01 0.01 0.01 0.01 0.5]
b: [0.01 0.5 0.5 0.5 0.5 0.5]
c: [0.01 0.01 0.01 0.5 0.5 0.5].

Confidence interval plots for these designs are given in figure 6. The best design for a given case is the one whose mean standard solution concentration is closest to the expected concentrations of the samples to be analysed. As a result, design a in figure 6 should be used for samples without iron contamination, while design b is suitable for contaminated samples. Design c is indicated for batches containing both kinds of samples.

Another relevant aspect of experimental design is the selection of the working range. The designs just compared have the same concentration range, but the program permits comparison of designs with different numbers of levels and different ranges.

In order to demonstrate the application of DESIGN, we return to the iron assay in drinking waters by ICP-AES. As already stated, in this analytical procedure the linear ranges are very wide [6] and the working range can be increased without risking deviation from linearity.

The following designs can be used to illustrate the use of option 3:

a: [0.01 0.01 0.01 0.5 0.5 0.5]
b: [0.01 0.01 0.01 0.01 1 1].

Results are shown in figure 7: design b provides a smaller confidence interval, when the whole range is considered (design a is better only in a small region, close to the concentration mean). This is due to the presence of the $\Sigma(x_i - x_m)^2$ sum in equation (1).

### Applications

A real example of iron determination in natural waters by atomic absorption spectrophotometry (AAS) follows. This illustrates the effect of some experimental designs discussed in this paper on the precision of estimated sample concentration [equation (3)].

Measurements were made using a Perkin-Elmer instrument, Model 503, following the procedure described in the manufacturer's manual. Standard solutions were prepared by dilution of Titrisol (MERCK) standards. Absorbances measured for each concentration level are presented in table 1.

In order to construct calibration curves, data from table 1 were used, according the following experimental designs:

(A) $X = [0.2\ \ 0.2\ \ 5\ \ 5]$

$x_m = 2.6$

$Y = [0.0084\ \ 0.0070\ \ 0.1794\ \ 0.1802]$.

(B) $X = [0.2\ \ 0.2\ \ 0.2\ \ 5\ \ 5\ \ 5]$

$x_m = 2.6$

$Y = [0.0078\ \ 0.0084\ \ 0.0068\ \ 0.1810\ \ 0.1802\ \ 0.1794]$.

(C) $X = [0.2 \quad 0.2 \quad 0.2 \quad 0.2 \quad 5 \quad 5 \quad 5 \quad 5]$

$x_m = 2.6$

$Y = [0.0068 \quad 0.0088 \quad 0.0070 \quad 0.0084 \quad 0.1794 \quad 0.1810 \quad 0.1802 \quad 1800]$.

(D) $X = [0.2 \quad 0.2 \quad 0.2 \quad 0.2 \quad 0.2 \quad 5]$

$x_m = 1$

$Y = [0.0088 \quad 0.0078 \quad 0.0068 \quad 0.0070 \quad 0.0084 \quad 0.1802]$.

(E) $X = [0.2 \quad 5 \quad 5 \quad 5 \quad 5 \quad 5]$

$x_m = 4.2$

$Y = [0.0078 \quad 0.1794 \quad 0.1800 \quad 0.1814 \quad 0.1802 \quad 0.1810]$.

(F) $X = [0.2 \quad 1 \quad 2 \quad 3 \quad 4.2 \quad 5]$

$x_m = 2.6$

$Y = [0.0078 \quad 0.0358 \quad 0.0732 \quad 0.1098 \quad 0.1520 \quad 0.1802]$.

(G) $X = [0.2 \quad 0.2 \quad 0.5 \quad 4.6 \quad 5 \quad 5]$

$x_m = 2.6$

$Y = [0.0078 \quad 0.0084 \quad 0.00178 \quad 0.1668 \quad 0.1802 \quad 0.1794]$.

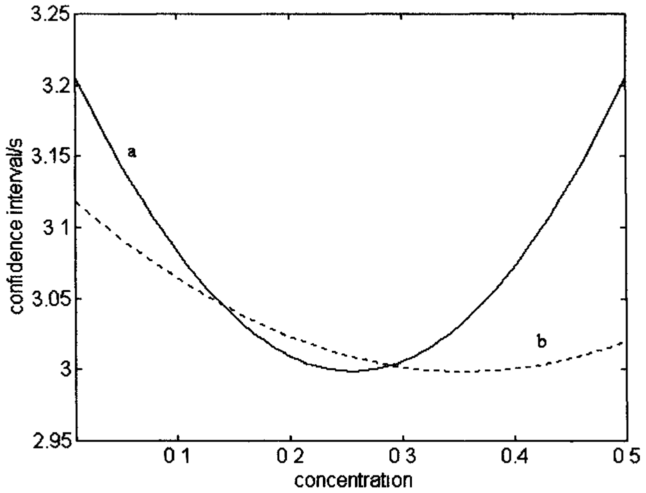

*Figure 7. Comparison of designs with different calibration ranges;* (a) *design* [0.01 0.01 0.01 0.5 0.5 0.5]; (b) *design* [0.01 0.01 0.01 0.01 1 1].

*Table 1. Absorbance values of standard solutions of iron. The repeated concentration values correspond to authentic replicates of the standard solutions series.*

| *Concentration (mg/l)* | *Absorbance* |
|---|---|
| 0.2 | 0.0078 |
| 0.2 | 0.0088 |
| 0.2 | 0.0068 |
| 0.2 | 0.0070 |
| 0.2 | 0.0084 |
| 0.5 | 0.0178 |
| 1.0 | 0.0358 |
| 2.0 | 0.0732 |
| 3.0 | 0.1098 |
| 4.2 | 0.1668 |
| 5.0 | 0.1794 |
| 5.0 | 0.1800 |
| 5.0 | 0.1814 |
| 5.0 | 0.1802 |
| 5.0 | 0.1810 |

*Table 2. Estimated values of the iron concentration (in mg/l) and 95% confidence interval in a sample of well water.*

| *Design* | *Estimated concentration* |
|---|---|
| A | $0.209 \pm 0.117$ |
| B | $0.209 \pm 0.071$ |
| C | $0.209 \pm 0.061$ |
| D | $0.209 \pm 0.068$ |
| E | $0.209 \pm 0.087$ |
| F | $0.209 \pm 0.075$ |
| G | $0.209 \pm 0.072$ |

It is important to note that the estimates of $b_1$ and $s$ in equation (3) are approximated to 0.0359 and 0.0008, respectively, for curves A–G above. Hence, confidence intervals will only depend on the terms related to experimental design [$t$, $n$ and $X$, in equation (3)].

The absorbance measured for a sample of well water was 0.0081 absorbance units. The estimated concentration and the 95% confidence intervals around this estimate for curves A–G are presented in table 2. Some of the features discussed in the previous section are highlighted by table 2:

(1) When the number of standard solutions increases from four (design A) to six (design B), the width of the confidence interval decreases 39% (from 0.117 to 0.071 mg/l). When the number of standard solutions goes from six to eight, the same interval decreases only by 14% (from 0.071 to 0.061 mg/l). This small decrease needs to be weighed against the task of preparing two more standard solutions to decide whether the increase in precision is worth the trouble.
(2) Designs B, F and G illustrate the effect of the number of concentration levels. Among these, the D-optimized design B presents the narrowest confidence interval, but design G, with four levels close to the limits of the working range, presents practically an equal interval (table 2).
(3) Designs D and E demonstrate how a shift in the average concentration ($x_m$) affects the precision of the calibration curve. With design D, $x_m = 1$ and with design E, $x_m = 4.2$. Because the estimated concentration $[(x_0)_1 = 0.209\,\text{mg/l}]$ is nearer to $x_m = 1$ than to $x_m = 4.2$, design D shows narrower confidence intervals for this estimate (0.068, table 2). This interval is slightly smaller than the D-optimized one (B), with the same number of standard solutions (0.071, table 2).

Experimental design, of course, varies from case to case and only the user is able to decide which design is the best for any particular situation.

### Conclusion

The DESIGN computational program presented in this work is conceived as a tool for the experimental design needed in building calibration curves. It is very simple to use and allows the experimenter to choose among several options.

It is hoped that this program will stimulate analytical chemists to adopt the practice of planning their experiments as a matter of routine.

### Acknowledgement

Partial support of CNPq is greatly appreciated.

### Appendix

```
%**SUBROUTINE FOR COMPUTATION OF CONFIDENCE INTERVALS AND EFFICIENCIES**
%X = Design vector
tam = length (X);
%****Computation of t-student (95% confidence)****
gral = (tam-2);
t5 = 2.282 ./(gral .^5);
t4 = 0.381 ./(gral .^4);
t3 = 2.9333 ./(gral .^3);
t2 = 2.77608 ./(gral .^2);
t1 = 2.37286 ./gral;
t = (1.95997 + t5 + t4 + t3 + t2 + t1);
%****Creating D-optimized design matrix ****
Xinic = min(X);
Xfin = max(X);
nm = round(tam/2);
for i = 1:nm
        Xotimt(i) = Xinic;
end
for i = (nm + 1):tam
        Xotimt(i) = Xfin;
end
%****Confidence intervals computation****
pass = abs((Xfin-Xinic) ./500);
Xotim  = Xotimt';
um = ones(size(Xotim));
Xotimaju = [um Xotim];
mato = Xotimaju'*Xotimaju;
invmato = inv(mato);
pontos = Xinic:pass:Xfin;
np = length(pontos);
Xajun = [um X];
matn = Xajun'*Xajun;
invmatn = inv(matn);
for i = 1:np
        Xo = [1 pontos(i)];
        intvoqst(i) = Xo*invmato*Xo';
        intvqstn(i) = Xo*invmatn*Xo';
          intvstn(i) = sqrt(1 + intvqstn(i));
        intvost(i) = sqrt(1 + intvoqst(i));
end
intvo = t .*intvost;
intvn = t .*intvstn;
%****Efficience computation****
Eficiencian = (det(matn) ./det(mato))*100;
```

### References

1. Aarons, L., 1981, *Analyst*, **106**, 1249.
2. Box, G. E. P., Hunter, W. G. and Hunter, J. S., 1987, *Statistics for Experiments* (New York: John Wiley).
3. Wolters, R. and Kateman, G., 1990, *Journal of Chemometrics*, **4**, 171.
4. Mermet, J. M., 1994, *Spectrochimica Acta*, **12–14**, 49B, 1313.
5. Pimentel, M. F. and Neto, B. B., 1996, *Química Nova*, **13**, 3, 268.
6. Draper, N. R. and Smith, H., 1981, *Applied Regression Analysis* (New York: John Wiley).
7. Montgomery, D. and Peck, E. A., 1982, *Introduction to Linear Regression Analysis* (New York: John Wiley).
8. St. John, R. C. and Draper, N. R., 1975, *Technnometrics*, **17**, 15.

